# Changing inequity in health service utilization and financial burden among patients with hypertension in China: evidence from China Health and Retirement Longitudinal Study (CHARLS), 2011–2018

**DOI:** 10.1186/s12939-023-02062-7

**Published:** 2023-11-24

**Authors:** Haoqing Tang, Mingyue Li, Larry Z. Liu, Yanbing Zhou, Xiaoyun Liu

**Affiliations:** 1 China Center for Health Development Studies, Peking University, Haidian District, 38 Xueyuan Road, Beijing, 100191 People’s Republic of China; 2 Department of Health Policy and Management, School of Public Health, Peking University, Beijing, China; 3 Center for Observational and Real-World Evidence (CORE), Merck & Co., Inc, Rahway, NJ 07065 USA; 4 Weill Cornell Medical College, New York City, NY USA; 5 MSD R&D (China) Co., Ltd, Shanghai, 200233 China

**Keywords:** Inequity, Affordability, Health service utilization, OOP payment, Catastrophic health expenditure, China

## Abstract

**Background:**

China initiated a health system reform in 2009 to achieve Universal Health Coverage (UHC) by 2020. While the effectiveness of health-system reforms has been studied, equity in health-service utilization and financial burden remains underexplored. This study evaluated whether the health system reform has improved the equity in utilization and financial burden of health services among patients with hypertension in China.

**Methods:**

We obtained data from four waves of the China Health and Retirement Longitudinal Study (CHARLS) conducted between 2011 and 2018. The main outcome variables were outpatient and inpatient service utilization rates and catastrophic health expenditure (CHE) for patients with hypertension. The Standardized Concentration Index (CI) was used to measure the changing equity in health service utilization and affordability.

**Results:**

Outpatient service utilization was relatively equal among patients with varying socioeconomic statuses (SESs) (CI: 0.041 in 2011 and 0.064 in 2018). Inpatient service utilization inequity improved from CI 0.144 in 2011 to CI 0.066 in 2018. CHE incidence increased from 15.6% in 2011 to 24.2% in 2018. CI for CHE declined from -0.069 in 2011 to -0.012 in 2015 but increased to -0.063 in 2018.

**Conclusions:**

Health insurance expansion and poverty alleviation policies promoted equity in inpatient service utilization for hypertensive patients. However, the financial burden for the poor requires further attention through reimbursement policy adjustments for outpatient services in primary care settings.

**Supplementary Information:**

The online version contains supplementary material available at 10.1186/s12939-023-02062-7.

## Introduction

Non-communicable diseases (NCDs), account for more than 70% of global mortality (41 million out of 58 million annual deaths) [1, 2]. The main type of NCD is cardiovascular disease (CVD), causing 17.9 million fatalities annually [3]. Hypertension, a primary risk factor for CVD, is a major cause of premature death globally, affecting an estimated 1.28 billion adults aged 30–79 years [4].

Poverty is closely linked to NCDs, with 77% of all NCDs deaths occurring in low- and middle-income countries. Socially disadvantaged groups are at a greater risk of exposure to tobacco or unhealthy eating habits, and having limited utilization of health services. Consequently, they are more likely to experience NCD-related morbidity and mortality. High medical expenses often push patients’ families into extreme poverty. To alleviate this problem, the United Nations (UN) established Sustainable Development Goals (SDGs) and committed to achieving universal health coverage (UHC) for all countries by 2030 [5]. UHC implies that all people should have access to quality health services without incurring financial hardship [6].

China is the world’s largest developing country. Since the 1980s, its economy and healthcare system have advanced rapidly. However, inequity in the access to and affordability of healthcare has become a serious concern. Owing to overreliance on out-of-pocket payments (OOPs) for healthcare financing, the incidence of catastrophic health expenditures (CHE) in China rose to 14% in 2008 [7]. Furthermore, this incidence was higher in the poorest of the population (approximately 20% in 2008) compared with the wealthiest of the population (approximately 10% in 2008) [8]. To address this issue, the Central Committee of the Communist Party of China and the State Council issued Opinions on Deepening Health System Reform in 2009 [9]. The reform covered a variety of measures, from strengthening primary healthcare (PHC) to expanding health insurance schemes, aiming to establish a basic healthcare system covering urban and rural residents by 2020 and create a healthcare system that is affordable, equitable, accessible, and effective [10]. By 2011, over 95% of the Chinese population was covered by health insurance [11]. However, this increase in health insurance coverage has not been translated into reductions in CHE among households [8].

To address health expenditure challenges among low-income people, China implemented Targeted Poverty Alleviation (TPA) in 2015 [12]. Till now, nearly 100 million rural residents have escaped poverty, benefiting from guarantees of adequate food, clothing, basic medical services, safe housing and compulsory education. China’s poverty alleviation movement should significantly contribute to the financial protection of the poor using health services, thereby promoting health equity [13].

Comprehensive evaluations have found positive trends in the access to and use of health services in China, but the trends of improvement in financial burden remain unclear. A 2003–2011 national study using the National Health Services Survey found that although the disparity in reimbursement rates between poor and wealthy households has decreased, poor households still face a greater financial burden [8]. Another nationally representative study using China Family Panel Studies (CFPS) data from 2010 to 2016 indicated that the rate of CHE declined, with the greatest decrease among households with the lowest SES [14]. However, another study using regional data from China suggested that the rate of CHE has not declined since 2011 (15.8% in 2013 and 16.5% in 2015) [5].

As equity is a central policy objective in China’s health reforms, consistent with the global principles of UHC and the SDGs [15], updated evidence is needed to better understand the levels and changing trends of equity in utilization and the financial burden of health services following China’s health system reform in 2009, particularly for patients diagnosed with NCDs. Recognizing that equity in access to health services may not mean equity in financial protection [16], this study investigates equity in both service utilization and financial protection and explores their relationship. The study interprets the changing trends of equity in access to and the financial burden of health services from the perspectives of health insurance and poverty alleviation.

## Methods

### Study design and data sources

#### Study design

Hypertension was used as a tracer to analyze changes in the inequity of outpatient and inpatient health service utilization and financial burden of patients in different socioeconomic groups and to explain the reasons for the changes in inequity from the perspective of medical insurance reimbursement and poverty alleviation policies.

#### Data sources

Data for this study were taken from the CHARLS, an continuous national survey of Chinese citizens aged 45 years and above from 28 provinces [17]. CHARLS uses multistage stratified probability-proportionate-to-size (PPS) selection to choose a nationally representative sample of Chinese citizens who are 45 years of age or older and conducts one-on-one interviews with them using a structured questionnaire. Demographic data, health status, healthcare use, medical insurance, family structure, and income are all covered by the questionnaire. The Peking University Biomedical Ethics Review Committee gave its clearance to this work (approval number: IRB00001052-11015). After reading a statement outlining the study’s goals, each responder gave their permission to proceed.

Four waves (2011, 2013, 2015, and 2018) of CHARLS data were obtained and analyzed in this study. With a sample size of 17,708 respondents, the first wave of CHARLS had an overall response rate of 80.5%. A follow-up survey was conducted every 2–3 years. To guarantee that the survey sample was nationally representative, the data included individual weighting variables. The CHARLS’s goals and procedures have been described in full elsewhere [17].

### Procedures

#### Hypertension

We defined hypertension as self-reported hypertension diagnosed by a doctor or self-reported use of antihypertensive medication. Households in which at least one person was diagnosed with hypertension were defined as hypertensive households.

#### Outcome variables

Outcome variables included health service utilization and financial burden. “Outpatient service utilization rate in the last month” and “inpatient service utilization rate in the last year” were used to measure health service utilization. Financial burden indicators included the proportions of OOPs and CHE. CHE was calculated using the methodology recommended by the WHO [18]. We defined a household as incurring CHE when OOP spending on health equaled or exceeded 40% of the household’s capacity to pay [19]. Household’s capacity to pay is defined as household non-subsistence spending. In other words, it refers to the financial ability of a household or family to afford expenses, payments, or taxes without experiencing undue financial hardship. Missing values for OOP household medical expenses were supplemented by calculating the sum of OOP outpatient and inpatient expenses for each household member. All expenditure-related variables were adjusted using the Consumer Price Index (CPI).

#### Independent variables

The independent variable in this study was SES. The annual per capita household consumption spending was used as a proxy for SES. We defined five socioeconomic groups based on the quintiles of per capita household consumption expenditure from the poorest to the richest.

Andersen’s behavioral model of health service use is one of the most acknowledged models of healthcare utilization in the literature [20]. In this study, the selection of covariates was guided by the theoretical framework of Andersen’s behavioral model, including predisposing characteristics, enabling factors, and need variables (Table 1).

**Table 1:** List of potential explanatory variables of health services utilization and financial burden grouped according to the Andersen's Behavioral Model of Health Services Use

	**Variables**	**Definition**
**Outcome variables**	**Health service utilization**	
	One-month outpatient visit	Visited a doctor in the last month; Yes = 1; No = 0
	One-year inpatient visit	Received inpatient care in the last year; Yes = 1; No = 0
	**Healthcare financial burden**	
	Proportion of OOP one-month outpatient payments	OOP payment (deducting the reimbursed expenses) / total payment for outpatient visits during the past month
	Proportion of OOP one-year inpatient payments	OOP payment (deducting the reimbursed expenses) / total payment for inpatient visits during the past year
	Catastrophic health expenditure	OOP spending on health equaled or exceeded 40% of a household’s capacity to pay
**Predisposing characteristics**	Age	If 45 = < age < 55, age = 1 If 55 = < age < 65, age = 2 If 65 = < age < 75, age = 3 If age > = 75, age = 4
	Gender	Male = 1; Female = 0
	Marital status	Married = 1; Single/divorced/widowed/Never married = 0
	Education	Primary school and below = 1; Secondary school = 2; College and above = 3
**Enabling factors**	Residence status	Agricultural household registration and living in rural areas = 1 Urban household registration and living in urban areas = 2 Agricultural household registration but living in urban areas = 3
	Health insurance	No insurance = 1 Urban employee basic medical insurance (UEBMI) = 2 Urban and rural resident basic medical insurance (URRBMI) = 3 Other insurance = 4
	Socioeconomic group	Per capita household consumption expenditure (PCE)
**Need variables**	Hypertension	①self-reported doctor diagnosed hypertension ②self-reported taking anti-hypertensive medication currently If ① = YES AND/OR ② = YES: With hypertension = 1 If ① = No AND ② = No: Without hypertension = 0
	Co-morbidity status	Participant with hypertension and one or more physical chronic non-communicable diseases = 1 Participant only with hypertension = 0

#### Statistical analysis

Pearson’s Chi-square tests were used to examine the differences in hypertension prevalence, healthcare utilization rates, and CHE incidence of patients with hypertension between groups, and t-test/F-test were used for continuous variables.

There are multiple methods available to measure socioeconomic-related inequity, such as the range method, Lorenz curve, and Gini coefficient. However, Concentration Curve (CC) and CI offer distinct advantages like displaying the share of health accounted for by cumulative proportions of individuals in the population ranked from poorest to richest and providing detailed information about specific percentiles. So, we used CC and CI to assess socioeconomic-related inequity in hypertension-care utilization and affordability.

The further the CC lies from the equality line (45-degree line), the higher the degree of inequity in healthcare and health service utilization and affordability [21].

The CI quantitatively reflects the degree of inequity. CI was calculated using the following equation:

$$CI = 2/\mu cov(h,r)$$

where *h* refers to the value of dependent variable in healthcare; *μ* is the mean of *h*; and *r* refers to the fractional rank of people according to the per capita household consumption expenditure (PCE), from the poorest to richest. A positive *CI* value indicates that service use is more likely among higher socioeconomic groups, while a negative *CI* value indicates that underprivileged people are more likely to use health services. A higher absolute value of *CI*indicates a higher degree of inequity, whereas a zero value indicates absolute equity [21].

Crude CI may be influenced by potential covariates. This study calculated covariate-standardized CI to control for confounding factors. Outcome variables were first standardized by covariates using the indirect standardization method as follows:

$${\widehat{y}}_{i}^{IS}={y}_{i}-{\widehat{y}}_{i}^{X}+\overline{y }$$

where ${\mathrm{y}}_{\mathrm{I}}$ is the actual value of the outcome variable, ${\widehat{y}}_{i}^{IS}$ is the predicted value of the outcome variable after running a probit/ordinary least squares model with exploratory variables (X), controlling for income and other socioeconomic factors included in the decomposition analysis, and $\overline{y }$ is the mean value of the outcome variable. This standardized outcome variable can be interpreted as the distribution of the expected outcome variable, regardless of how the covariates are distributed across socioeconomic factors.

All statistical analyses were conducted using STATA (version 17.0; Stata Corp., College Station, TX, USA). *P* values less than 0.05 were considered statistically significant.

### Patient and public involvement

Patients and the public were not involved in this methodological research. This research was based on a second hand data (CHARLS).

## Results

### Descriptive statistics

Table 2 presents the demographic characteristics of the study participants. The mean age of all participants was 59.1 years (SD = 9.9) in 2011, which increased to 61.7 years (SD = 10.1) in 2018. Most of the participants were female (51.2–52.8%), married or partnered (80.1–85.1%), had primary education or below (66.2–67.9%), and resided in rural areas (55.2–58.1%). Approximately 20% of the participants were rural to urban migrants. The majority of participants (91.4–97%) had at least one type of health insurance. The most popular scheme was the URRBMI, covering 73.5–79.4% of total population. The percentage of participants without any insurance decreased from 6.6% in 2011 to 3.0% in 2018.

**Table 2:** Demographic characteristics and hypertension prevalence of study population in China, 2011–2018 [n(%) /mean(sd)]

Variables	Demographic Characteristics and hypertension prevalence							
	**2011 wave**		**2013 wave**		**2015 wave**		**2018 wave**	
	**Total**	**Hypertension**	**Total**	**Hypertension**	**Total**	**Hypertension**	**Total**	**Hypertension**
Total	16,970	4283 (25.2%)	17,557	5180 (29.5%)	16,102	4810 (29.9%)	19,732	6496 (32.9%)
Age (years)								
45–54	6144 (36.2%)	1046 (17.0%)	5801 (33.0%)	1155 (19.9%)	5398 (33.5%)	960 (17.8%)	5665 (29.6%)	1168 (20.6%)
55–64	6282 (37.0%)	1666 (26.5%)	6513 (37.1%)	1959 (30.1%)	5496 (34.1%)	1668 (30.3%)	6260 (32.7%)	2033 (32.5%)
65–74	3087 (18.2%)	1039 (33.7%)	3542 (20.2%)	1386 (39.1%)	3590 (22.3%)	1466 (40.8%)	4935 (25.8%)	2091 (42.4%)
75 and above	1457 (8.6%)	532 (36.5%)	1701 (9.7%)	680 (40.0%)	1618 (10.0%)	716 (44.3%)	2285 (11.9%)	1063 (46.5%)
${\chi }^{2}$		439.3***		505.2***		742.8***		775.9***
Sex								
Male	8288 (48.8%)	1959 (23.6%)	8504 (48.4%)	2403 (28.3%)	7770 (48.3%)	2206 (28.4%)	9304 (47.2%)	3037 (32.6%)
Female	8682 (51.2%)	2324 (26.8%)	9053 (51.6%)	2777 (30.7%)	8332 (51.7%)	2604 (31.3%)	10,428 (52.8%)	3459 (33.2%)
${\upchi }^{2}$		22.0***		12.3***		15.7***		0.6
Marital status								
Married or Partnered	13,586 (80.1%)	3364 (24.8%)	14,206 (80.9%)	4097 (28.8%)	13,023 (80.9%)	3764 (28.9%)	16,800 (85.1%)	5285 (31.5%)
Unmarried and Others	3383 (19.9%)	918 (27.1%)	3351 (19.1%)	1083 (32.3%)	3078 (19.1%)	1045 (34.0%)	2932 (14.9%)	1211 (41.3%)
${\upchi }^{2}$		8.1**		15.8***		30.3***		109.6***
Education								
Primary school and below	11,301 (66.6%)	2908 (25.7%)	11,615 (66.2%)	3554 (30.6%)	10,919 (67.9%)	3300 (30.2%)	12,923 (65.5%)	4413 (34.1%)
Secondary school	5302 (31.3%)	1263 (23.8%)	5585 (31.8%)	1511 (27.1%)	4824 (30.0%)	1397 (29.0%)	6383 (32.3%)	1949 (30.5%)
College and above	361 (2.1%)	108 (29.9%)	355 (2.0%)	114 (32.1%)	343 (2.1%)	113 (32.9%)	426 (2.2%)	134 (31.5%)
${\upchi }^{2}$		11.3**		24.0***		4.1		25.7***
Residence status								
Rural	9774 (57.6%)	2241 (22.9%)	10,065 (57.5%)	2777 (27.6%)	8424 (55.2%)	2418 (28.7%)	11,054 (58.1%)	3516 (31.8%)
Urban	3814 (22.5%)	1250 (32.8%)	4060 (23.2%)	1445 (35.6%)	3656 (24.0%)	1231 (33.7%)	3966 (20.8%)	1491 (37.6%)
Rural-to-urban	3369 (19.9%)	789 (23.4%)	3390 (19.4%)	944 (27.8%)	3185 (20.9%)	873 (27.4%)	4005 (21.1%)	1316 (32.9%)
${\upchi }^{2}$		148.3***		94.5***		39.6***		44.4***
Health insurance								
None	1119 (6.6%)	243 (21.7%)	681 (3.9%)	178 (26.1%)	1331 (8.6%)	390 (29.3%)	593 (3.0%)	169 (28.5%)
UEBMI	1746 (10.3%)	587 (33.6%)	2034 (11.7%)	724 (35.6%)	1567 (10.1%)	527 (33.6%)	2549 (12.9%)	934 (36.6%)
URRBMI	13,129 (77.7%)	3165 (24.1%)	13,825 (79.4%)	3939 (28.5%)	11,416 (73.5%)	3355 (29.4%)	15,277 (77.4%)	4965 (32.5%)
Others	904 (5.3%)	270 (29.9%)	875 (5.0%)	287 (32.8%)	1228 (7.9%)	372 (30.3%)	1311 (6.6%)	426 (32.5%)
${\upchi }^{2}$		91.5***		51.4***		12.6**		22.6***
Socioeconomic group								
Quintile1 (lowest)	2855 (20.0%)	671 (23.5%)	2326 (20.0%)	669 (28.8%)	2183 (20.0%)	638 (29.2%)	3301 (19.7%)	1025 (31.1%)
Quintile2	2855 (20.0%)	649 (22.7%)	2326 (20.0%)	663 (28.5%)	2182 (20.0%)	629 (28.8%)	3411 (20.4%)	1112 (32.6%)
Quintile3	2855 (20.0%)	726 (25.4%)	2329 (20.0%)	633 (27.2%)	2182 (20.0%)	585 (26.8%)	3427 (20.5%)	1108 (32.3%)
Quintile4	2854 (20.0%)	735 (25.8%)	2327 (20.0%)	690 (29.7%)	2182 (20.0%)	669 (30.7%)	3378 (20.2%)	1171 (34.7%)
Quintile5 (highest)	2853 (20.0%)	820 (28.7%)	2322 (20.0%)	711 (30.6%)	2181 (20.0%)	647 (29.7%)	3224 (19.3%)	1098 (34.1%)
${\upchi }^{2}$		33.1***		7.5		8.5		12.5*

^***^ *p* < 0.001

^**^ *p* < 0.01

^*^p < 0.05 significance test

Table 2 also presents the prevalence of hypertension in the study population from 2011 to 2018. Overall, the prevalence of hypertension among adults aged 45 years and over in China increased from 25.2% in 2011 to 32.9% in 2018. The prevalence of hypertension increased with increasing socioeconomic status: 23.5% individuals in the lowest socioeconomic quintile and 28.7% in the highest quintile in 2011 were diagnosed with hypertension versus 31.1% and 34.1%, respectively, in 2018. The proportion of people with co-morbidity status was around 20%-30% in 2011–2018 (Table S1).

### Health service utilization and financial burden of patients with hypertension

Table 3 displays changes in health service utilization rates of patients diagnosed with hypertension. The percentage of outpatient visits among patients with hypertension remained relatively stable between 2011 and 2018 (23.0% in 2011; 25.4% in 2013; 22.4% in 2015; and 20.4% in 2018).

**Table 3:** Changes in healthcare service utilization rate of people with hypertension in China, 2011–2018 [n (%)]

Variables	healthcare service utilization rate							
	**One-month outpatient service utilization rate**				**One-year inpatient service utilization rate**			
	**2011 wave**	**2013 wave**	**2015 wave**	**2018 wave**	**2011 wave**	**2013 wave**	**2015 wave**	**2018 wave**
Total	966 (23.0%)	1289 (25.4%)	1057 (22.4%)	1298 (20.4%)	620 (14.5%)	993 (19.2%)	955 (19.9%)	1543 (24.3%)
Socioeconomic group								
Quintile1 (lowest)	128 (19.3%)	153 (23.2%)	120 (18.9%)	179 (17.5%)	56 (8.4%)	95 (14.2%)	91 (14.3%)	194 (18.9%)
Quintile2	161 (25.1%)	155 (23.8%)	146 (23.5%)	204 (18.3%)	81 (12.5%)	97 (14.6%)	93 (14.8%)	251 (22.6%)
Quintile3	164 (22.9%)	148 (23.8%)	131 (22.6%)	194 (17.5%)	97 (13.4%)	106 (16.8%)	116 (19.8%)	251 (22.7%)
Quintile4	178 (24.7%)	187 (27.5%)	140 (21.5%)	261 (22.3%)	113 (15.4%)	149 (21.6%)	148 (22.2%)	326 (27.9%)
Quintile5 (highest)	176 (22.1%)	193 (28.1%)	153 (24.4%)	268 (24.4%)	168 (20.5%)	172 (24.2%)	158 (24.4%)	292 (26.6%)
${\upchi }^{2}$	8.1	7.6	6.6	27.6 ***	47.8 ***	35.6 ***	33.2 ***	30.6 ***

^***^p < 0.001

^**^p < 0.01

^*^p < 0.05 significance test

The outpatient service utilization rate increased with increasing SES. Chi-square tests revealed that the outpatient visit rate in the lowest socioeconomic quintile in 2018 (17.5%) was significantly lower than the highest quintile (24.4%; *p* < 0.001). The percentage of inpatient service utilization among patients with hypertension increased from 14.5% in 2011 to 24.3% in 2018. The inpatient service utilization rate also increased with increasing SES. Chi-square tests demonstrated that the inpatient service utilization rate was higher in the highest quintile than in the lowest quintile from 2011 to 2018 (*p* < 0.001), with 8.4% in the lowest socioeconomic quintile and 20.5% in the highest quintile in 2011, compared with 18.9% and 26.6%, respectively, in 2018.

Table 4 presents healthcare expenses and the incidence of CHE in patients with hypertension.

**Table 4:** Changes in health care expenses and CHE incidence of people with hypertension in China, 2011–2018

Variables	Health care expenses and CHE incidence																			
	**Outpatient service [mean]**								**Inpatient service [mean]**								**CHE incidence [n (%)]**			
	**Total expense (CNY)**				**% of OOP**				**Total expense (CNY)**				**% of OOP**							
	**2011**	**2013**	**2015**	**2018**	**2011**	**2013**	**2015**	**2018**	**2011**	**2013**	**2015**	**2018**	**2011**	**2013**	**2015**	**2018**	**2011**	**2013**	**2015**	**2018**
Total	271.0	400.4	516.8	359.7	86.9	82.6	81.9	79.7	1416.0	2298.5	2857.1	4195.2	62.7	55.9	53.1	51.2	479 (15.6%)	511 (18.2%)	534 (19.9%)	1111 (24.2%)
Socioeconomic group																				
Quintile1 (lowest)	78.8	163.7	308.7	125.3	89.8	85.4	87.9	81.1	466.8	1002.5	901.1	1259.9	68.6	54.1	54.2	46.4	95 (17.1%)	103 (18.8%)	101 (19.2%)	221 (27.0%)
Quintile2	119.4	184.2	263.0	343.4	92.7	83.6	83.2	80.7	798.5	945.4	1085.3	2462.0	68.7	59.0	56.8	51.2	100 (17.4%)	102 (17.9%)	104 (19.3%)	252 (27.2%)
Quintile3	229.0	350.9	239.7	257.8	90.2	84.0	86.7	81.1	671.7	1009.3	2201.5	2608.4	69.7	55.3	49.5	51.0	113 (17.9%)	103 (18.9%)	118 (23.3%)	247 (26.2%)
Quintile4	251.1	489.5	466.6	411.7	88.0	84.1	80.1	77.3	911.8	2234.3	3583.8	4492.0	63.5	55.3	53.2	49.4	104 (16.6%)	104 (18.0%)	133 (23.2%)	234 (23.6%)
Quintile5 (highest)	629.8	914.5	1157.0	766.2	76.7	75.5	78.8	76.8	3800.9	5659.9	6210.9	10,053.6	54.8	55.0	53.2	51.9	67 (9.9%)	99 (17.4%)	78 (14.7%)	157 (17.2%)
${\upchi }^{2}$/ F	6.1***	5.8***	9.7***	6.5***	10.4***	3.4**	2.7*	1.0	28.2***	26.9***	17.6***	19.9***	4.7**	0.3	0.6	1.2	21.8***	0.6	17.0**	34.6***

^***^p < 0.001

^**^p < 0.01

^*^p < 0.05 significance test

The total expense of outpatient payments increased from CNY 271.0 in 2011 to CNY 516.8 in 2015 and decreased in 2018 (CNY 359.7). The proportion of OOP for outpatient services also decreased from 86.9% in 2011 to 79.7% in 2018. The proportion of OOP for outpatient services by different insurance types was around 80%-90% and for inpatient services was around 50%-60% (Table S2). The mean total expense for outpatient payments increased with higher SES, reaching CNY 766.2 in the highest socioeconomic quintile and CNY 125.3 in the lowest quintile, in 2018.

The total expense of inpatient payment increased from CNY 1216.0 in 2011 to CNY 4195.2 in 2018. The proportion of OOP for inpatient services decreased from 62.7% in 2011 to 51.2% in 2018. The mean total expense of inpatient payments increased with increasing SES, reaching CNY10053.6 in the highest socioeconomic quintile and CNY1259.9 in the lowest socioeconomic quintile in 2018.

A growing trend in the incidence of CHE was observed between 2011 and 2018. The incidence of CHE in all households in 2011, 2013, 2015, and 2018 was 15.6%, 18.2%, 19.9%, and 24.2%, respectively. Except for the 2015 wave, chi-square tests indicated that CHE incidence among patients with hypertension was higher among rural adults than among urban adults (*p* < 0.001). The rural–urban gap was 4.8% in 2011, 6.0% in 2013, 4.1% in 2015, and 8.6% in 2018, suggesting that the gap had widened between 2011 and 2018. Except for the 2013 wave, chi-square tests showed that CHE incidence was higher among patients in the lowest quintile than among those in the highest quintile in 2011 (*p* < 0.001) but lower in 2018 (*p* < 0.001). The incidence of CHE was 27.0% in the lowest socioeconomic quintile and 17.2% in the highest socioeconomic quintile in 2018, indicating that the poorer the household was, the higher the incidence of CHE.

### Changes in inequity of healthcare utilization of patients with hypertension

**Fig. 1:**
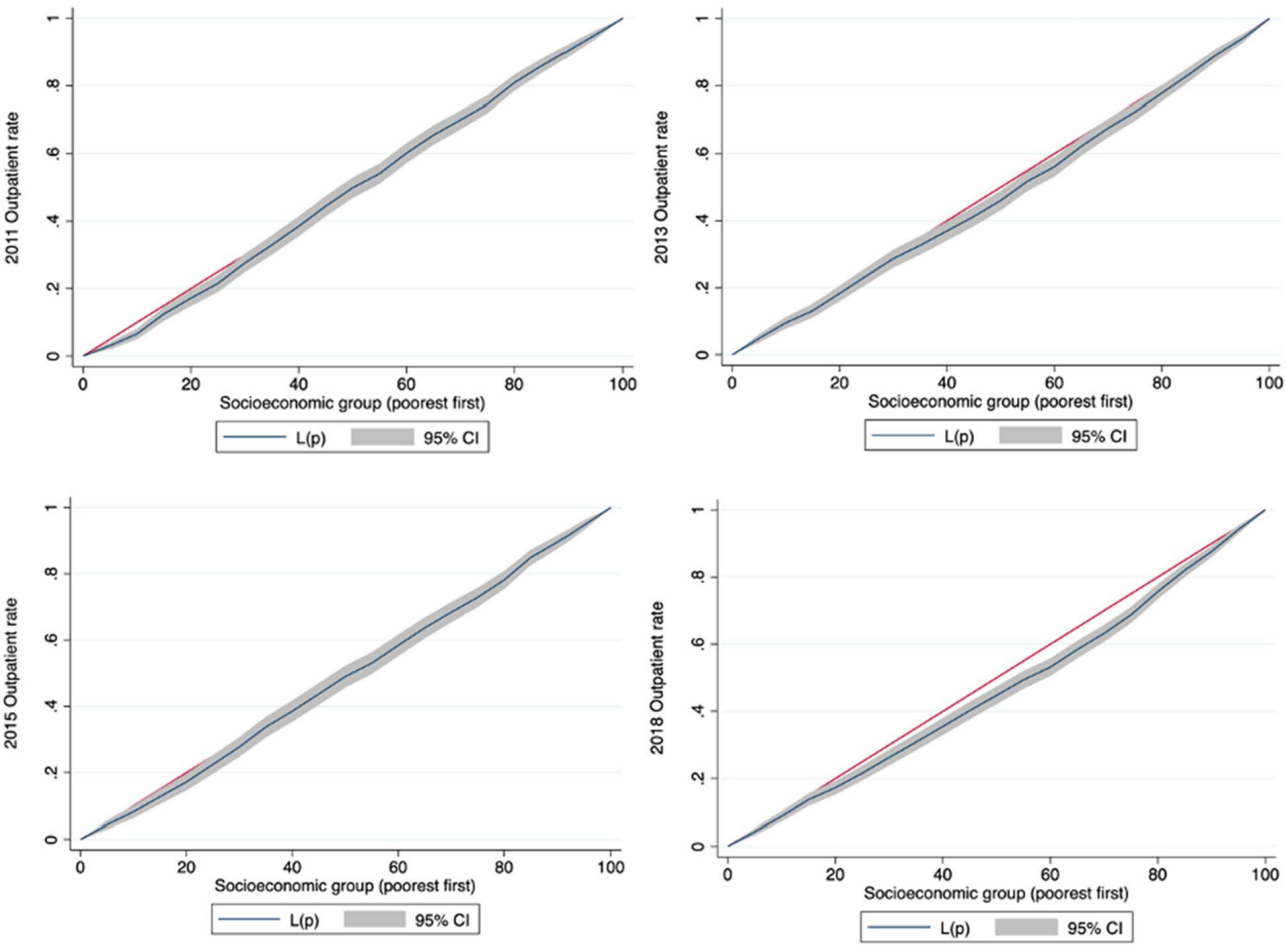
Concentration curves of outpatient utilization rate for people with hypertension in China in 2011–2018

Table 5 displays the results of the socioeconomic-related inequity analysis for the healthcare utilization rate among patients with hypertension. Outpatient service utilization was relatively equal among patients from different socioeconomic groups from 2011 to 2018. The CC for outpatient visits almost overlapped with the equality line during this period (Fig. 1).

**Table 5:** Standardized healthcare utilization rate for hypertensive people in China across socioeconomic group and concentration indexes [% / mean]

	**Standardized healthcare utilization rate**							
	**Outpatient**				**Inpatient**			
	**2011 Wave**	**2013 Wave**	**2015 Wave**	**2018 Wave**	**2011 Wave**	**2013 Wave**	**2015 Wave**	**2018 Wave**
Socioeconomic group								
Quintile1 (lowest)	18.59	22.78	19.98	18.13	8.76	13.77	13.71	19.08
Quintile2	24.26	23.96	23.03	18.65	12.63	14.55	15.14	22.83
Quintile3	22.09	23.66	22.34	18.24	13.58	17.09	18.85	23.11
Quintile4	25.22	27.56	20.59	21.92	15.27	21.76	21.82	27.98
Quintile5 (highest)	23.57	28.72	25.25	24.19	19.73	24.69	24.20	26.17
Ratio (highest/lowest)	1.27	1.26	1.26	1.33	2.25	1.79	1.77	1.37
Deviation (highest-lowest)	4.98	5.94	5.27	6.06	10.97	10.92	10.49	7.09
Unstandardized CI	0.018	0.039*	0.029	0.075***	0.163***	0.128***	0.114***	0.070***
Standardized CI	0.041*	0.046**	0.024	0.064***	0.144***	0.139***	0.115***	0.066***

^***^p < 0.001

^**^p < 0.01

^*^p < 0.05 significance test

**Fig. 2:**
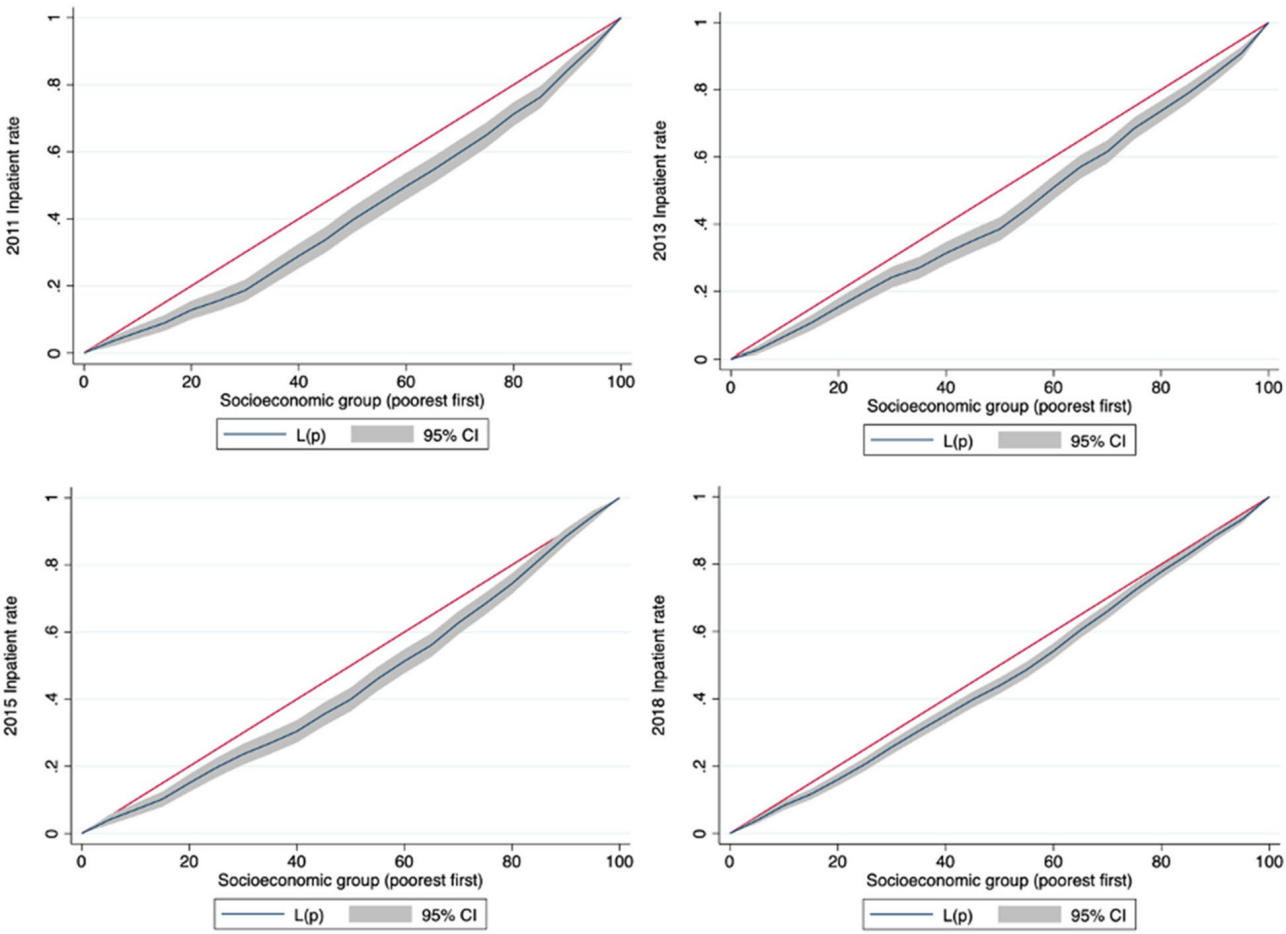
Concentration curves of inpatient utilization rate for people with hypertension in China in 2011–2018

The inpatient utilization rate gap between the wealthiest and most deprived quintiles narrowed between 2011 and 2018. In 2011, the proportion of inpatient visits in the wealthiest quintile of people with hypertension was 2.25 times higher than that in the most deprived quintile in China. This ratio was reduced 1.37 times in 2018. The CI for inpatient service utilization was 0.144 in 2011, considerably higher than that for outpatient service utilization (0.041). This finding implies that wealthier individuals use more inpatient services than poorer individuals. Inpatient service utilization equity greatly improved greatly from 2011 to 2018. The CI for inpatient service utilization decreased from 0.144 in 2011 to 0.066 in 2018. These changing trends were also observed in CC (Fig. 2).

### Changes in inequity of OOP outpatient and inpatient payments and CHE incidence of people with hypertension

**Fig. 3:**
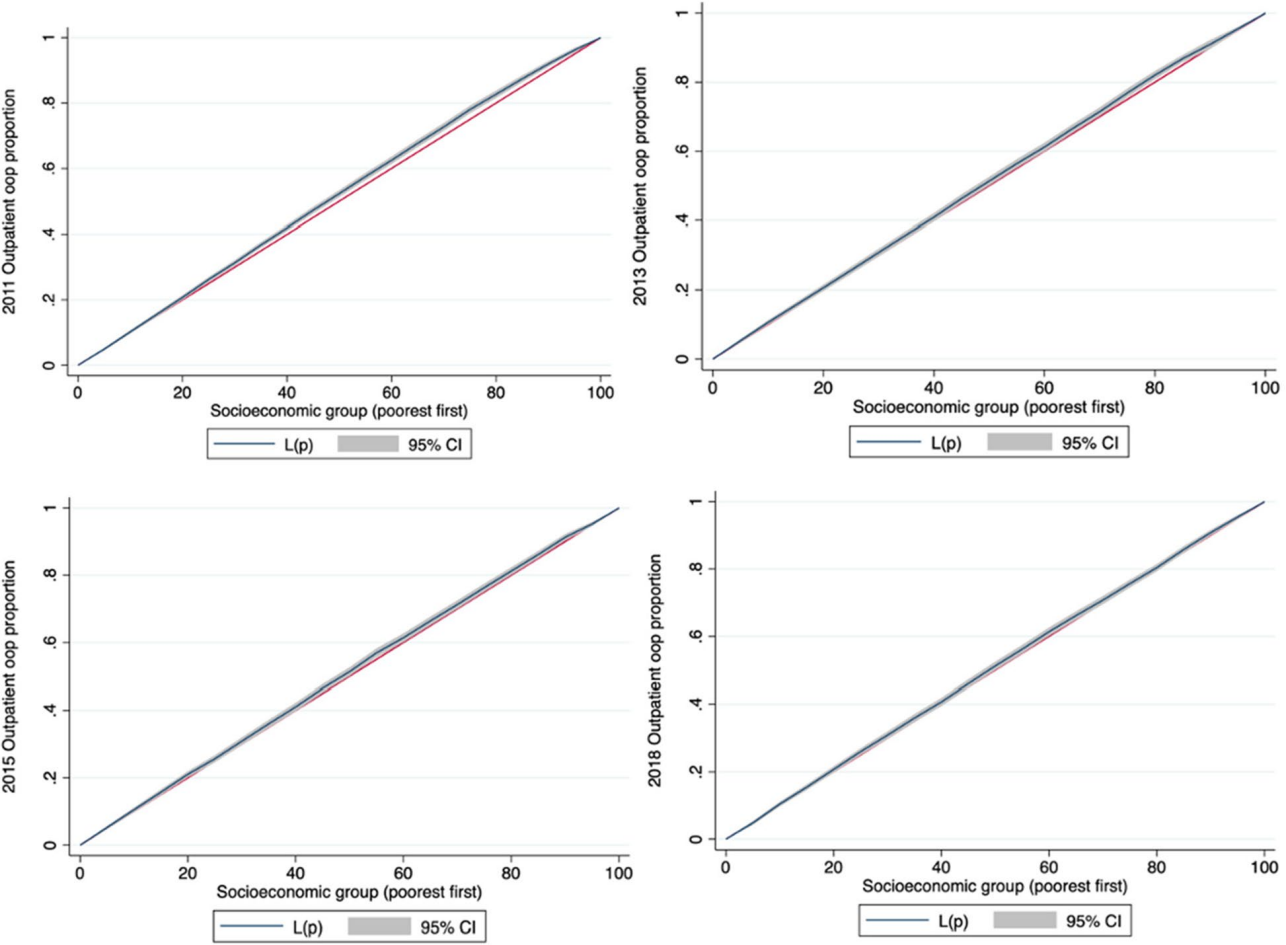
Concentration curves of proportion of out-of-pocket outpatient payments for people with hypertension in China in 2011–2018

Table 6 presents the results of the socioeconomic-related inequity analysis for the proportion of OOP outpatient and inpatient payments and CHE incidence among patients with hypertension. The proportion of OOP outpatient payments was relatively equal among patients from different socioeconomic groups from 2011 to 2018. The CC for the proportion of OOP outpatient payments almost overlapped with the equality line in 2011–2018 (Fig. 3).

**Table 6:** Standardized proportion of out-of-pocket outpatient and inpatient payments and CHE incidence for hypertensive people in China across socioeconomic group and concentration indexes

	**Standardized % of OOP payments[mean]**								**Standardized CHE incidence**			
	**Outpatient**				**Inpatient**							
	**2011 Wave**	**2013 Wave**	**2015 Wave**	**2018 Wave**	**2011 Wave**	**2013 Wave**	**2015 Wave**	**2018 Wave**	**2011 Wave**	**2013 Wave**	**2015 Wave**	**2018 Wave**
Socioeconomic group												
Quintile1 (lowest)	85.79	83.04	85.52	76.95	62.64	49.68	46.66	43.67	16.58	17.67	18.23	25.69
Quintile2	89.92	82.41	80.25	77.81	63.84	55.91	56.03	49.01	17.04	17.27	19.08	26.44
Quintile3	88.27	83.49	85.26	80.16	65.04	54.63	49.38	50.01	17.78	18.76	23.63	26.11
Quintile4	88.04	83.01	81.06	78.49	62.77	54.54	54.28	50.22	16.79	18.44	23.27	24.18
Quintile5 (highest)	83.34	78.98	82.9	81.38	62.26	59.97	55.86	54.65	10.67	18.75	15.41	18.6
Ratio (highest/lowest)	0.97	0.95	0.97	1.06	0.99	1.21	1.20	1.25	0.64	1.06	0.85	0.72
Deviation (highest-lowest)	-2.45	-4.06	-2.62	4.43	-0.38	10.29	9.2	10.98	-5.91	1.08	-2.82	-7.09
Unstandardized CI	-0.034***	-0.021**	-0.020**	-0.015*	-0.059***	-0.005	-0.009	0.013	-0.084***	-0.005	-0.028	-0.084***
Standardized CI	-0.009	-0.007	-0.001	0.008	-0.009	0.031*	0.023	0.035***	-0.069**	0.023	-0.012	-0.063***

^***^p < 0.001

^**^p < 0.01

^*^p < 0.05 significance test

**Fig. 4:**
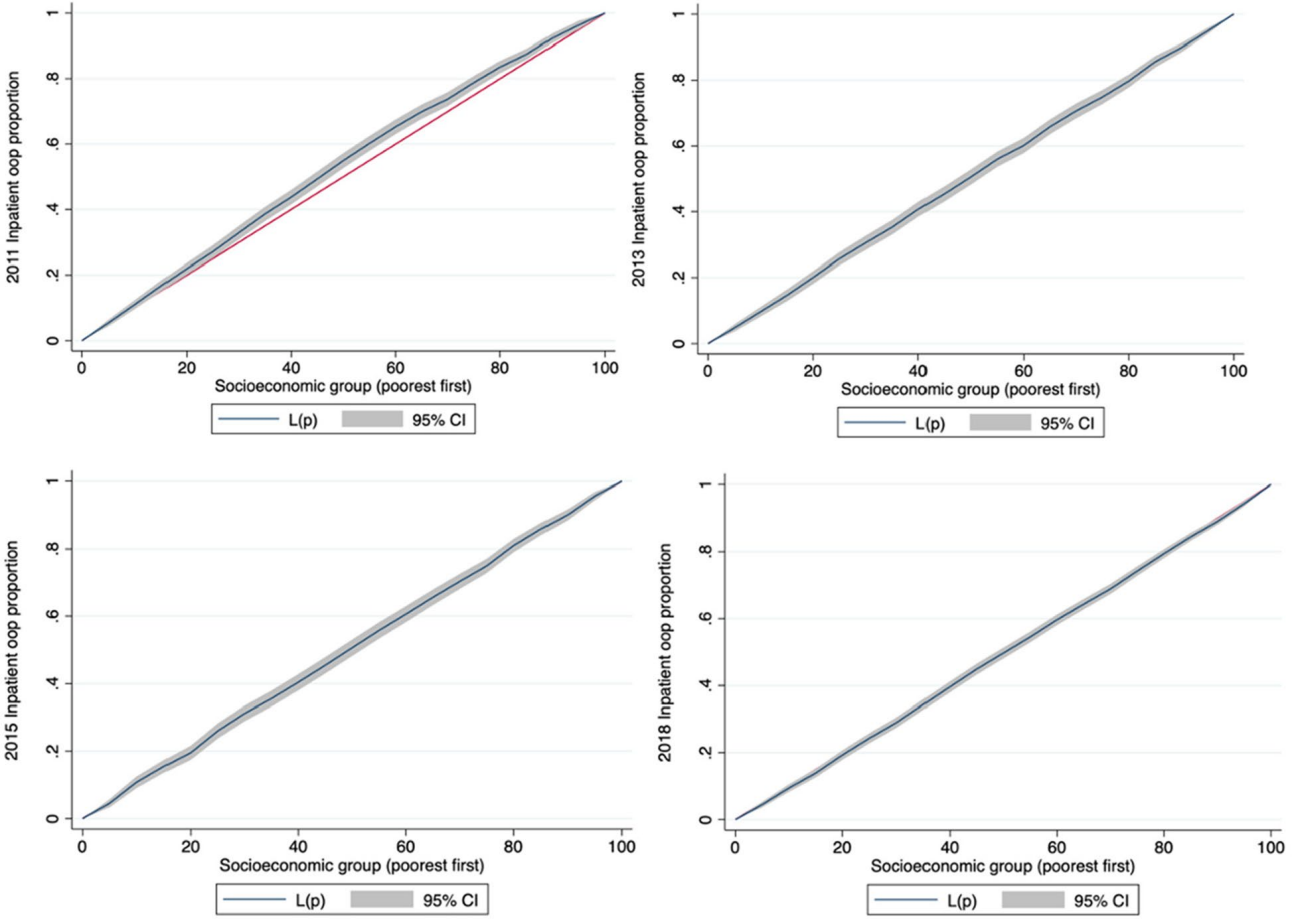
Concentration curves of proportion of out-of-pocket inpatient payments for people with hypertension in China in 2011–2018

The proportion of OOP for inpatient services was also relatively equal among patients from different socioeconomic group between 2011 and 2018. The CI changed from -0.009 in 2011 to 0.031 in 2018. Meanwhile, the CC for the proportion of OOP inpatient payments almost overlapped with the equality line in 2011–2018 (Fig. 4).

**Fig. 5:**
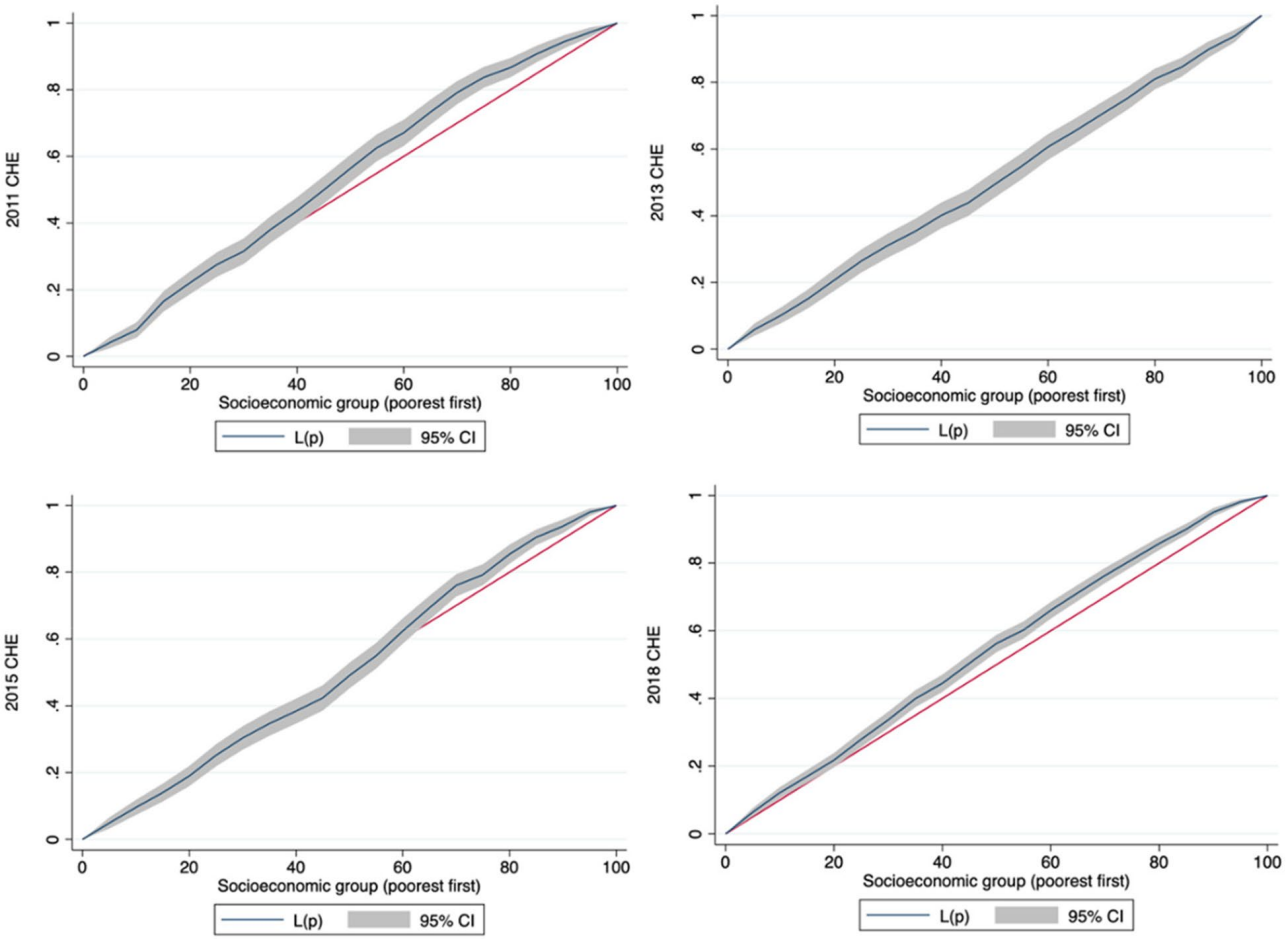
Concentration curves of CHE incidence for people with hypertension in China in 2011–2018

Regarding the affordability of hypertension, equity in CHE exhibited a complex, changing trend. It decreased in 2013 and 2015 but increased in 2018. The CC for the incidence of CHE was above the equality line in 2011 and almost overlapped with the equality line in 2013 and 2015. However, it was significantly higher than the equality line in 2018. The CI decreased from -0.069 (2011) to -0.012 (2015) but increased to -0.063 (2018) (Fig. 5).

## Discussion

China is experiencing a heavy disease burden of hypertension, which accounted for 14.28% of disability-adjusted life-years and 27.5% of deaths in 2013 [22]. This study used hypertension as a tracer disease to analyze the level and changing trends of inequity in healthcare utilization and financial burden from 2011 to 2018. China’s health system reform has achieved substantial improvements in equity in inpatient-service utilization for patients with hypertensive patients. However, patients’ financial burden has continued to increase, with the occurrence of an increasing CHE from 15.6% in 2011 to 24.2% in 2018. The equity in financial burden improved from 2011 to 2015 but then showed a reverse trend in 2018.

Health insurance and poverty-alleviation policies have significantly contributed to equity improvements in inpatients-service utilization. In contrast to the market-oriented approach of the previous two decades, the 2009 health system reform prioritized equity and created a universal health insurance system [8]. From 2009 to 2015, China expanded the coverage of basic health insurance schemes, raised the level of benefit packages, improved urban and rural medical assistance systems, and enhanced basic health insurance management and services. From 2015 to 2020, China improved its financing sustainability and reimbursement rate adjustment for basic health insurance, deepened the reform of provider payment methods, promoted the integration of basic health insurance schemes, improved the protection mechanism for catastrophic illnesses, and advanced the development of commercial insurance. After over a decade of considerable effort, health insurance schemes have ensured coverage of almost the entire Chinese population. In 2018, RMB723.2 per capita funding was allocated to the URRBMI was [7], accounting for 4.95% of the per capita net income of rural residents. In parallel, the share of OOP expenditures out of total healthcare expenditures dropped to 29.27% in 2015, halving the share from 2001 (59.97%) [23]. High levels of insurance coverage and reimbursement promote equity in health service utilization and reduce the proportion of OOP.

To promote financial protection for the poor to use health services, in 2015, China launched a health poverty alleviation program to address the problem of health expenditure for low-income individuals and complete poverty alleviation by 2020 [24]. Catastrophic Disease Insurance has become an important means of alleviating poverty induced by poor health and favors poor households. In 2013, over 200 million people participated in Catastrophic Disease Insurance and the Catastrophic Disease Insurance Scheme (CDIS) was fully implemented in China [25]. Universal pension coverage is also a key mechanism for promoting equity in accessing health services. China began developing a rural pension scheme in 2009. Almost all rural residents, including the poor, were enrolled in the scheme [26]. The evidence shows that the rural pension scheme has significantly contributed to poverty alleviation in rural areas [27].

Although health insurance schemes and poverty alleviation policies have increased reimbursement rates and reduced the share of OOP payments for both outpatient and inpatient services, patients in different socioeconomic groups face an unequal financial burden. Poor people still faced a higher financial burden measured by CHE, compared to the non-poor people.

The current incidence of CHE in China remains one of the highest in the world [28–31]. However, there has been controversy regarding the changing trend of financial burden due to health service utilization in China since the 2009 health system reform. Previous studies have found that the incidence of CHE in China is declining [14, 32]. For example, one study based on CFPS showed that in 2010, 2012, 2014, and 2016, the CHE rates in China were 13.58, 11.95, 11.43 and 11.06%, respectively [14]. However, other studies have reached the opposite conclusion [24, 33]. These controversies may arise from different methodologies, target populations, or time periods used in the studies. Our study found that patients with hypertension suffered an increase in CHE from 15.6% in 2011 to 24.2% in 2018. We focused on patients aged 45 and older who were diagnosed with hypertension; therefore, the CHE rate may have been higher than that in the general population.

Our study found that equity in CHE increased from 2011 to 2015. A possible reason maybe the healthcare system reform, the rural pension scheme and CDIS have greatly increased the universal basic health insurance coverage, the income of impoverished populations and reduced the likelihood of medical impoverishment [16] However, the equity in CHE decreased in 2015–2018. This increasing inequity of financial burden may be attributed to the improvement in the utilization rate of medical services and OOP expenditure for NCDs. We found that health insurance provided a higher reimbursement rate for inpatient services (approximately 48.8% in 2018) than outpatient services (approximately 20.3% in 2018), which is consistent with previous studies [34, 35]. Nevertheless, certain earlier researches have indicated a notably elevated reimbursement rate for both outpatient and inpatient services [36, 37]. These divergent findings could potentially be attributed to variations in the data sources (medical institution data vs. household survey data) and the specific study cohorts (general population vs. middle-aged and elderly population aged 45 and above) used in the studies. Simultaneously, the health poverty alleviation program significantly enhanced hospitalization reimbursement rate for the most impoverished individuals. This encouraged a higher inclination among impoverished individuals to opt for hospital-based medical services, particularly inpatient care, rather than utilizing primary care facilities (From 2015–2018, the hospitalization rates for the lowest quintile increased 5.37% while the highest quintile only increased 1.97%). Additionally, since impoverished individuals tend to reside in regions with limited healthcare resources, the quality of outpatient care is generally lower. Consequently, this has led to increased hospitalization rates for NCDs, and also increasing the incidence of CHE among impoverished populations and eroding healthcare equity. On the other hand, the prevalence of hypertension among the most impoverished individuals has significantly increased, thus, to some extent, adding to the financial burden and also increased the inequality of CHE.

Hypertension is widely regarded as a disease that can be managed through quality out-of-hospital services [38–41]. This increasing financial burden may be related to the increased use of inpatient services, especially in the poorest group. Although health insurance has lowered the threshold for the utilization of inpatient services, it still incurs extra medical expenses compared with outpatient services, thereby increasing the financial burden on households with chronic diseases. In light of this, future policymaking should focus more on providing PHC services to vulnerable groups and adjusting the reimbursement policy favoring appropriate service to reduce avoidable hospitalization and relieve the financial burden.

This study had several limitations. First, the results of this study may be interpreted as a reflection of the overall impact of all reform measures as well as social developments during the period 2011–2018. We could not isolate the independent effects of any specific policy over the past few years. Second, all the data were self-reported, which may have introduced a reporting bias. Third, we did not have data regarding indirect medical costs (e.g., accommodation and transportation) or opportunity costs (e.g., financial loss from job absence or decreased productivity), leading us to underestimate the real cost of medical expenses in China.

In conclusion, China’s health system reform has improved the equity in inpatient utilization among patients diagnosed with hypertension. However, patients still face an increasing financial burden owing to the increasing use of inpatient services, especially in the poorest group.

Considering that most patients with hypertension need only outpatient services rather than inpatient services, health insurance schemes and future poverty alleviation efforts should focus more on outpatient services in primary care settings and provide a higher reimbursement rate for outpatient services to guide patients’ healthcare seeking habits. This would result in scarce resources for impatient services for those with complicated and severe conditions.

### Supplementary Information

**Additional file 1:** **Table S1.** Demographic Characteristics of patient with co-morbidity status in China, 2011-2018 [n(%) /mean(sd)]. **Table S2.** Changes in health care expenses of patients with different insurance types in China, 2011-2018.

## Data Availability

The datasets generated and analyzed during the current study are publicly available in the CHARLS website [http://charls.pku.edu.cn].

## Abbreviations

UHC: Universal Health Coverage
CHARLS: China Health and Retirement Longitudinal Study
CHE: Catastrophic health expenditure
CI: Concentration Index
NCD: Non-communicable disease
CVD: Cardiovascular diseases
UN: United Nations
SDGs: Sustainable Development Goals
OOPs: Out-of-pocket
PHC: Primary health care
UEBMI: Urban Employee Basic Medical Insurance
URRBMI: Urban and Rural Residents Basic Medical Insurance
TPA: Targeted Poverty Alleviation
CFPS: China Family Panel Studies
CPI: Consumer price index
CC: Concentration Curve
PCE: Per capita household consumption expenditure
CDIS: Catastrophic Disease Insurance scheme
