# Supplementary material for: Changing inequity in health service utilization and financial burden among patients with hypertension in China: evidence from China Health and Retirement Longitudinal Study (CHARLS), 2011–2018
Source: Int J Equity Health. 2023 Nov 24;22:246. doi: 10.1186/s12939-023-02062-7 (PMC10668495; doi:10.1186/s12939-023-02062-7)
Supplement: Supplementary file 1 — Additional file 1: Table S1. Demographic Characteristics of patient with co-morbidity status in China, 2011-2018 [n(%) /mean(sd)]. Table S2. Changes in health care expenses of patients with different insurance types in China, 2011-2018. [file 12939_2023_2062_MOESM1_ESM.docx]

**Changing inequity in health service utilization and financial burden among patients with hypertension in China: Evidence from China Health and Retirement Longitudinal Study (CHARLS), 2011–2018**

*Supplementary file*

**Table S1** Demographic Characteristics of patient with co-morbidity status in China, 2011-2018 [n(%) /mean(sd)]

| **Variables** | **Co-morbidity status** | | | |
| --- | --- | --- | --- | --- |
|  | **2011 wave** | **2013 wave** | **2015 wave** | **2018 wave** |
| Total | 3161 (18.6%) | 3691 (21.0%) | 3705 (23.0%) | 5500 (27.9%) |
| Age (years) |  |  |  |  |
| 45-54 | 736 (12.0%) | 761 (13.1%) | 664 (12.3%) | 914 (16.1%) |
| 55-64 | 1258 (20.0%) | 1423 (21.8%) | 1301 (23.7%) | 1712 (27.3%) |
| 65-74 | 785 (25.4%) | 1017 (28.7%) | 1183 (33.0%) | 1838 (37.2%) |
| 75 and above | 382 (26.2%) | 490 (28.8%) | 557 (34.4%) | 920 (40.3%) |
| $\chi^{2}$ | 336.9*** | 409.2*** | 670.2*** | 774.4*** |
| Sex |  |  |  |  |
| Male | 1428 (17.2%) | 1678 (19.7%) | 1656 (21.3%) | 2505 (26.9%) |
| Female | 1733 (20.0%) | 2013 (22.2%) | 2049 (24.6%) | 2995 (28.7%) |
| $\chi^{2}$ | 20.9*** | 16.6*** | 24.4*** | 8.9** |
| Marital status |  |  |  |  |
| Married or Partnered | 2494 (18.4%) | 2911 (20.5%) | 2885 (22.2%) | 4443 (26.4%) |
| Unmarried and Others | 666 (19.7%) | 780 (23.3%) | 819 (26.6%) | 1057 (36.1%) |
| $\chi^{2}$ | 3.2 | 12.7*** | 27.9*** | 114.5*** |
| Education |  |  |  |  |
| Primary school and below | 2191 (19.4%) | 2591 (22.3%) | 2586 (23.7%) | 3764 (29.1%) |
| Secondary school | 889 (16.8%) | 1024 (18.3%) | 1035 (21.5%) | 1621 (25.4%) |
| College and above | 78 (21.6%) | 76 (21.4%) | 84 (24.5%) | 115 (27.0%) |
| $\chi^{2}$ | 18.5*** | 35.9*** | 9.8** | 29.7*** |
| Residence status |  |  |  |  |
| Rural | 1651 (16.9%) | 1973 (19.6%) | 1864 (22.1%) | 2969 (26.9%) |
| Urban | 944 (24.8%) | 1047 (25.8%) | 971 (26.6%) | 1304 (32.9%) |
| Rural-to-urban | 564 (16.7%) | 662 (19.5%) | 645 (20.3%) | 1101 (27.5%) |
| $\chi^{2}$ | 121.7*** | 72.3*** | 43.3*** | 53.6*** |
| Health insurance |  |  |  |  |
| None | 176 (15.7%) | 121 (17.8%) | 289 (21.7%) | 141 (23.8%) |
| UEBMI | 436 (25.0%) | 510 (25.1%) | 401 (25.6%) | 808 (31.7%) |
| URRBMI | 2333 (17.8%) | 2815 (20.4%) | 2606 (22.8%) | 4181 (27.4%) |
| Others | 203 (22.5%) | 208 (23.8%) | 295 (24.0%) | 368 (28.1%) |
| $\chi^{2}$ | 67.7*** | 32.1*** | 8.0* | 25.5*** |
| Socioeconomic group |  |  |  |  |
| Quintile1 (lowest) | 470 (16.5%) | 467 (20.1%) | 478 (21.9%) | 822 (24.9%) |
| Quintile2 | 476 (16.7%) | 463 (19.9%) | 476 (21.8%) | 949 (27.8%) |
| Quintile3 | 536 (18.8%) | 448 (19.2%) | 447 (20.5%) | 929 (27.1%) |
| Quintile4 | 553 (19.4%) | 517 (22.2%) | 539 (24.7%) | 1012 (30.0%) |
| Quintile5 (highest) | 624 (21.9%) | 526 (22.7%) | 498 (22.8%) | 962 (29.8%) |
| $\chi^{2}$ | 36.9*** | 13.0** | 12.2* | 29.0*** |

*** p < 0.001, ** p < 0.01, * p < 0.05 significance test.

Co-morbidity status: In this study, we defined Co-morbidity status as participant with hypertension and one or more self-reported diagnosed physical non-communicable diseases (diabetes, dyslipidaemia, heart disease, stroke, cancer, chronic lung disease, digestive disease, liver disease, kidney disease, and arthritis). We did not include individuals with self-reported psychiatric and memory-related diseases due to potential recall bias.

**Table S2** Changes in health care expenses of patients with different insurance types in China, 2011-2018

| **Variables** | **Outpatient service [mean]**  **% of OOP** | | | | **Inpatient service [mean]**  **% of OOP** | | | |
| --- | --- | --- | --- | --- | --- | --- | --- | --- |
|  | **2011 wave** | **2013 wave** | **2015 wave** | **2018 wave** | **2011 wave** | **2013 wave** | **2015 wave** | **2018 wave** |
| **Total** | 86.9 | 82.4 | 82.2 | 79.5 | 62.7 | 55.9 | 53.0 | 51.2 |
| Health insurance |  |  |  |  |  |  |  |  |
| None | 98.0 | 95.7 | 83.8 | 93.5 | 87.6 | 88.2 | 59.1 | 73.0 |
| UEBMI | 63.3 | 64.2 | 66.4 | 63.5 | 41.5 | 38.1 | 36.0 | 41.7 |
| URRBMI | 90.6 | 85.6 | 85.0 | 83.9 | 70.1 | 60.0 | 57.1 | 53.2 |
| Others | 74.7 | 71.4 | 74.7 | 66.1 | 39.9 | 45.6 | 46.8 | 48.0 |
